# Tonsillitis and Its Treatment

**Published:** 1883-11-20

**Authors:** L. B. Dawley

**Affiliations:** New York


### TONSILLITIS AND ITS TREATMENT.

BY L. B. DAWLEY, M. D., OF N. Y.

Without going into the minutiæ or the symptomatology of the very common disease called quinsy, which every medical man in active practice is called upon to treat, I beg leave to give my experience :

An attack of tonsillitis is usually ushered in with a feeling of malaise, headache, pains in the limbs, and a sense of chilliness, or by a rigor. There is a sense of uneasiness in the throat, the patient complaining of soreness there, with lancinating pains extending toward the ears ; and at or behind the angle of the jaws very much tenderness and stiffness exist, so that the patient may be unable to open the mouth wide enough for an examination to be made. The expression of the face is one of much suffering and anxiety. The tonsils are much swollen, and fluids regurgitate through the nose on attempting to swallow. The mouth is usually filled with a yellow, tenacious mucous saliva ; and this may assume the form of a membrane, which I have removed, in some cases, in large masses, with my dressing forceps. The patient rarely assumes the recumbent position, owing to the great sense of suffocation.

Secretions are arrested, pulse increased, elevation of temperature, the voice assuming a nasal-twang, and in very bad cases there will be loss of voice, which very much frightens the patient and friends. There is usually much prostration, and this is due to the inability of the patient to take and digest nourishment.

I think if the cases are seen early, that they may terminate in resolution, avoiding abscesses and suppuration. All of my cases, and some of them of the worst form, have terminated in this way.

CASE I.—Maud E., aged 20 years, small in stature, of general good health, was taken with usual symptoms of quinsy, July 11, 1882. She called in a homœopathist of many years' practice and quite noted in throat troubles. Prescribed his usual high dilutions and inhalations of hot steam. Case grew worse under his treatment, till the family concluded to change. They called me in July 18th. I found the patient bolstered up in a large chair with a teapot in her lap and a mush poultice on her throat. Patient said she had not slept a minute, nor spoke a word, nor eaten a mouthful, in a week. (Informed me by writing on a slate.) I opened her mouth with tongue spatula, and with dressing forceps removed much yellow, tenacious mucus from fauces. Gave—

℞ Tr. aconite.................................... gtt. x,

Tr. phytolacca.................................. ʒ j.

Water........................................... ℥ iv.

Dose, teaspoonful half an hour apart.

Applied to tonsils with camel's hair brush, every hour, tincture of veratrum and tincture muriate of iron, *aa.* ʒ ij. Patient began spitting freely. I applied tincture veratrum (strong) to the outside of the throat by wetting a cloth and keeping it in contact with the neck. Saw patient next day, and found her able to take nourishment in liquid form. Continued treatment, and discharged my patient the third day.

The above case came to me some time afterward with symptoms pointing to the same disease. I duplicated the treatment, and the patient had no further trouble.

CASE II.—Mrs. D., aged 35 years, strong, healthy constitution. I was called to see her October 2d, 1882. She said she was subject to quinsy attacks, and always had her throat lanced, or was confined to the house for ten days at a time:

℞ Aconite.......................................gtt. x,

Phytolacca.......................................ʒ ij,

Water.......................................℥ iv.

M. S. A teaspoonful every half hour.

Local application of veratrum.......................................ʒ j,

Tinc. muriat of iron.......................................ʒ j.

Water.......................................℥ ij.

M. S. Use in throat with atomizer once every hour. Case terminated in resolution.

CASE III.—Chas. M., aged 28 years, large, strong, and in the best of health, with the exception of occasional attacks of quinsy. He came to my office February 18th, 1883, and with the nasal twang which plainly spoke for itself; said he had been the rounds of the doctors, and could get no relief. He concluded to see what I could do. I prescribed—

℞ Aconite.......................................gtt. x,

Phytolacca.......................................ʒ j,

Veratrum.......................................gtt xx,

Water.......................................℥ iv.

M. S. A teaspoonful every hour.

Had a cloth wet in spirits of turpentine and applied to the throat. Saw him a week afterwards, when he met me and said: "Doctor, you did what no other doctor ever accomplished." Said he went to bed after using my medicine, and slept all night, and next day could take food in liquid form. No suppuration.

CASE IV.—Miss C., aged 18 years, presented herself at my office May 9th, 1883, and had the same story to tell of what she had heard of my success in curing quninsy. She was just coming down with the disease. I prescribed as follows:

℞ Aconite.......................................gtt. x,

Phytolacca.......................................ʒ j,

Veratrum.......................................gtt. xx,

Water.......................................℥ iv.

M. S. A teaspoonful every hour.

Applied spirits of turpentine to the throat as in previous case. Patient came back in a week, and said that she was quite well. Suppuration had always occurred on previous attacks.

I could go on and multiply cases, but I think this is sufficient. My success with phytolacca in glandular diseases has been quite extended. In the treatment of chronic tonsillitis I rely solely upon phytolacca and tonics.—*Eclec. Med. Jour.*

